# Combined use of CDAI and blood indices for assessing endoscopic activity in ileocolic Crohn’s disease

**DOI:** 10.1186/s12876-023-02968-0

**Published:** 2023-09-28

**Authors:** Xiaolin Hu, Jiajia Li, Yunyun Sun, Dacheng Wu, Tiantian Zhao, Maofeng Ma, Jie Chen, Mei Wang, Sicong Hou

**Affiliations:** 1 Department of Gastroenterology, Affiliated Hospital of Yangzhou University, Yangzhou, 225009 Jiangsu China; 2 Medical College of Yangzhou University, Jiangsu, China; 3 Department of Gastroenterology, Northern Jiangsu People’s Hospital Affiliated to Yangzhou University, Yangzhou, 225009 Jiangsu China

**Keywords:** Crohn’s disease, CDAI, Blood indices, SES-CD, Endoscopic activity

## Abstract

**Background:**

Mucosal healing has become the primary treatment target for patients with Crohn’s disease (CD). We aimed to develop a noninvasive and convenient tool to evaluate the endoscopic activity in patients with ileocolic CD.

**Methods:**

A retrospective multicenter study including 300 CD patients (training, 210 patients; test, 90 patients) was conducted at two tertiary referral centers. Independent risk factors associated with endoscopic activity were explored, which were then combined into a comprehensive index. The predictive performance was evaluated with the area under receiver operating characteristic curve (ROC). Cohen’s Kappa was adopted to examine the consistency between each indicator and endoscopic activity.

**Results:**

A total of 210 CD patients were recruited in the training cohort. We found that Crohn’s Disease Activity Index (CDAI), C-reactive protein (CRP) and platelet-to-lymphocyte percentage ratio (PLpR) were independently associated with endoscopic activity. Additionally, the comprehensive index generated from the above three indices achieved good discrimination and performed better than CDAI in AUC (0.849 vs. 0.769, *P* < 0.05). This was further well demonstrated by the external test cohort, which showed good discrimination (AUC: 0.84, 95% CI: 0.744–0.936). Intra-individual comparison revealed the comprehensive index to be superior in the prediction of endoscopic activity. In the subgroup analysis, the AUC of comprehensive index was significantly higher than CDAI especially in inflammatory phenotype (0.824 vs. 0.751, *P* < 0.05).

**Conclusion:**

Combining CDAI, CRP and PLpR significantly improved the accuracy for predicting endoscopic activity in ileocolic CD, which can help better monitor an endoscopic flare.

**Supplementary Information:**

The online version contains supplementary material available at 10.1186/s12876-023-02968-0.

## Introduction

Crohn’s disease (CD) is a chronic inflammatory disorder which affects the whole gastrointestinal tract with symptoms involving abdominal pain, chronic diarrhea and weight loss [1]. As a worldwide disease, the prevalence and incidence of CD has rapidly increased [2], particularly in Asian countries [3]. Moreover, some multinational population-based studies revealed that the incidence of CD in China ranged from 0.07 to 3.86 per 100,000 people per year [4], which was especially higher in the coastal areas [5]. Although some CD patients have purely inflammatory when disease diagnosed, many will subsequently progress to develop stricturing and/or penetrating disease over time [6]. Therefore, effective monitoring of gastrointestinal inflammation is crucial for clinical decision-making and ultimately preventing complications and reducing long-term disability.

The Selecting Therapeutic Targets in Inflammatory Bowel Disease (STRIDE) consensus defined clinical remission as a short- or intermediate-term target and endoscopic healing as a long-term therapeutic target of CD [7, 8]. A ‘treat-to-target’ strategy encouraged dynamically optimizing therapy according to the regular assessment of staged targets. Endoscopic remission plays a vital role in mucosal healing which has been proved to be associated with lower relapse rates, hospitalization rates and reduced need for surgery [9, 10]. Although mucosal healing has emerged as the primary treatment target for patients with CD, a significant limitation of adoption of it as routine measure is the reliance on colonoscopy which is invasive, expensive, and repeated endoscopic procedures is not practical nor acceptable to patients [11]. Therefore, it is crucial to find a non-invasive surrogate index to evaluate endoscopic activity.

Crohn’s Disease Activity Index (CDAI) has been most widely used in clinical practice and trials to assess the symptomatic response or remission. However, its subjective nature and poor correlation with endoscopic activity limited its clinical application. The CALM trial is an open-label multicenter phase 3 randomized controlled trial which enrolled 244 patients with active endoscopic CD who were randomized to tight control or clinical management groups. In clinical management group, patients had a therapy escalation if CDAI decrease of < 100 points or CDAI ≥ 200, while in tight control group, a therapy escalation was conducted when fecal calprotectin ≥ 250 µg/g, C-reactive protein (CRP) ≥ 5 mg/L and CDAI ≥ 150. It turned out that compared with clinical management group, patients in tight control group presented better mucosal healing with absence of deep ulcers, deep remission, biological remission and steroid-free remission. These results suggested that the optimization of therapy based on combination of clinical symptoms and laboratory parameters was more likely to achieve better endoscopic and clinical outcomes than conventional symptoms-based decision [12]. Consistently, several clinical studies have attempted to investigate the performance of composite index including physical symptoms and objective biomarkers, but till now no practical surrogate algorithm can evaluate the endoscopic manifestations [13].

Various markers for CD have been identified in blood, stool, urine and colonic tissue over the past decades. Despite the fecal indicators, such as calprotectin and lactoferrin have been considered as efficient tools for discriminating endoscopic activity, there were still several limitations in clinical application. Previous studies showed that compliance of about two-thirds of the patients with calprotectin was poor [14], and there is significant sample variability depending on the time of stool collection [15]. Urinary intestinal fatty acid-binding protein (I-FABP) has been shown to be a potential index of disease activity in patients with CD [16]. Besides, urinary tricarboxylic acid cycle intermediates, such as citrate and succinate, also implicated in predicting endoscopic activity and remission [17], however, the urinary indices has not been widely used in clinical practice and needs further investigation. Moreover, endoscopic disease activity has been shown to be evaluated by the degree of epithelial damage and inflammation, mononuclear cell infiltration within the lamina propria, neutrophils within the epithelium, the presence of vesicles/ulcers and granulomas [18]. Although colonic tissue index mentioned above has become standard tools for evaluating disease activity, the primary limitations are the reliance on repeat colonoscopy and expert knowledge in pathology. The blood-based indices are the most widely used in monitoring disease activity due to its convenience and economy, however, the results are far from satisfactory. To date, some laboratory parameters have been proved to be associated with clinical activity, such as hemoglobin (HB), red blood cell distribution width (RDW), platelet, mean platelet volume (MPV), albumin [19–21]. Meanwhile, several indirect indicators including neutrophil-to-lymphocyte ratio (NLR), platelet-to-lymphocyte ratio (PLR), platelet-to-lymphocyte percentage ratio (PLpR), CRP-to-albumin ratio (CRP/ALB) platelet-to-albumin ratio (PLT/ALB) were demonstrated to be correlated with endoscopic activity [22–24]. Considering that individual index is usually lack of specificity or sensitivity, some researchers focused on the combination of diverse serum indices to enhance the effectiveness in evaluating CD activity. However, to our knowledge, no ideal predictive indicator is an alternative to endoscopy in daily clinical practice.

The present study was designed to identity objective and noninvasive serum indicators on the basis of CDAI to assess endoscopic disease activity in CD patients. We believe that combined use of clinical symptoms and laboratory serum indices has great potential to develop as surrogate markers of endoscopic activity.

## Methods

### Study design and patients

This retrospective multicenter cohort study included 300 patients who were diagnosed with CD and underwent hematology and endoscopy examination from 2 tertiary referral centers from January 2015 to January 2023. The training cohort was recruited from the Affiliated Hospital of Yangzhou University and the external test cohort enrolled patients from Northern Jiangsu People’s Hospital Affiliated to Yangzhou University. This study was approved by the Research Ethics Committee of the Affiliated Hospital of Yangzhou University [No. (2021-YKL06-09-006)].

The inclusion criteria were as follows: (1) the diagnosis CD on the basis of clinical symptoms, laboratory examinations, endoscopic findings, histological results and imaging data; (2) endoscopy and laboratory examination data must be available concurrently, and the results of laboratory examination were obtained within 7 days before colonoscopy. The exclusion criteria were: (1) limited to upper gastrointestinal or small intestinal CD; (2) history of intestinal resection; (3) coexistence of other autoimmune diseases (e.g., ankylosing spondylitis, systemic lupus erythematosus, Sjogren’s syndrome); (4) hematologic diseases or other diseases which influence the results of the complete blood counts (e.g., systemic or intestinal infections); (5) pregnancy or lactation.

### 2.2. Clinical data collection and collation

Data including age, gender, age at diagnosis, disease duration, body mass index (BMI), Montreal classification, CDAI score, endoscopic and radiological results, and therapeutic methods were achieved from the electronic medical record system. The result relating to hematological indicators including whole blood count, CRP and albumin were collected from the clinical laboratory system. Furthermore, the indirect indicators were calculated (e.g., NLR, PLpR, CRP / ALB).

### Endoscopic data and outcomes

Endoscopic disease activity was evaluated by the Simple Endoscopic Score for CD (SES-CD) which divides the intestinal segment into five parts: terminal ileum (including ileocecal valve), right colon, transverse colon, left colon (including sigmoid colon) and rectum [25]. The severity of each intestinal segment includes four variables: size of ulcers (none, diameter 0.1-0.5 cm, 0.5-2 cm, > 2 cm), ulcerated surface (none, < 10%, 10-30%, > 30%), affected surface (none, < 50%, 50-75%, > 75%) and presence of narrowing (none; single, can be passed; multiple, can be passed; cannot be passed). The scores of each variable range from 0 to 3. According to consensus, endoscopic remission was defined as SES-CD of 0–2, mild endoscopic activity 3–6, moderate 7–15, and severe > 15. Two senior endoscopists performed endoscopic scoring individually. When they faced conflicting scores, a consistent scoring discussed by them was regarded as the final score. To avoid bias, the endoscopists were blinded to the other results (e.g., values of laboratory parameters and CDAI).

### Statistical analysis

#### Sample size evaluation

A sample size of at least 34 endoscopic procedures (17 endoscopic disease remission and 17 endoscopic disease activity) was required in the training and test cohorts according to the following hypothesis: power, 90%; two-sided significance level, 0.05; alternative hypothesis of the AUC, 0.8 compared with the null hypothesis of the AUC, 0.5, and the allocation of the positive group was equal to that of negative group. Therefore, sample sizes of 210 (54 endoscopic disease remission and 156 endoscopic disease activity) in the training cohort and 90 (21 endoscopic disease remission and 69 endoscopic disease activity) in the test cohort were sufficient to detect an AUC difference of 0.5 with 90% power if the true AUC was > 0.8. Statistical analyses were performed using PASS (version 2021).

#### Predictive performance

Non-normally distributed data were expressed as median (interquartile range, IQR) and compared with Mann-Whitney U test between different groups of endoscopic activity. Categorical data were described as frequencies and compared using Chi-square test and fisher exact test. The Spearman correlation coefficient was used to express the correlation between indices and SEC-CD. Logistic regression by stepwise regression was performed to explore the independent risk factors associated with endoscopic activity which were then combined into a new composite index. The receiver operating characteristic (ROC) curve was used to describe the ability of each index to predict endoscopic activity and to determine the cut-off value (according to Youden index). The calculation of sensitivity, specificity, positive predictive value (PPV), negative predictive value (NPV), accuracy was based on the cut-off values and corresponding computational formulas. The prediction efficacies were evaluated by calibration. Cohen’s Kappa were adopted to examine the consistency between each indicator and endoscopic activity, and Delong’s test was used for comparison of prediction ability among indicators. A *P*-value < 0.05 was considered as significant. Statistical analysis was performed in SPSS version 25.0 (IBM, Armonk, NY) and R version 4.1.1 (R Foundation for Statistical Computing, Vienna, Austria).

## Results

### Patient characteristics

A total of 210 CD patients (118 males and 92 females; median age: 38 years; IQR: 27–49 years) were enrolled in training cohort. The demographic and clinical characteristics are shown in Table 1. In regard to the Montreal classification, 117 cases (55.7%) were diagnosed at the age of 17 to 40 years old and the most common disease location was ileocolic (61.4%). As for disease phenotype, there were almost equal numbers of people in inflammatory (44.8%) and structuring (44.3%) behavior. A total of 85 (40.5%) patients had a history of perianal disease. Most patients were treated with biologics (40.0%), followed by immunosuppressants (35.7%), 5-ASA (21.4%), No medication (14.3%) and corticosteroids (10.5%). A total of 90 CD patients with a median age of 37.5 years were enrolled in the external test cohort.

**Table 1:** Comparison of Demographic and Clinical Characteristics Between Training and Test Cohorts

Characteristics	Training Cohort (n = 210)	Test Cohort (n = 90)	*P* value
Age at assessment (in years), median (IQR)	38 (27–49)	37.5 (26-51.25)	0.14
Disease duration, years, median (IQR)	1 (0–4)	2 (1–6)	0.065
Gender			0.919
Male, n (%)	118 (56.2)	50 (55.6)	
Female, n (%)	92 (43.8)	40 (44.4)	
BMI, median (IQR)	20.8 (18.8–22.6)	20.4 (18-22.4)	0.18
Age at diagnosis (in years), n (%)			0.925
A1 (≤ 16)	17 (8.1)	8 (8.9%)	
A2 (17–40)	117 (55.7)	48 (53.3)	
A3 (> 40)	76 (36.2)	34 (37.8)	
Disease location, n (%)			0.144
L1 (terminal ileum)	46 (21.9)	23 (25.6)	
L2 (colon)	12 (5.7)	11 (12.2)	
L3 (ileocolon)	129 (61.4)	50 (55.6)	
L3 + L4 (L3 + upper gastrointestinal tract)	23 (11)	6 (6.7)	
Disease phenotype, n (%)			0.475
B1 (inflammatory)	94 (44.8)	40 (44.4)	
B2 (stricturing)	93 (44.3)	44 (48.9)	
B3 (penetrating)	23 (11)	6 (6.7)	
Medication, n (%)			< 0.05
No medication	30 (14.3)	8 (8.9)	
5-ASA	45 (21.4)	21 (23.3)	
Corticosteroids	22 (10.5)	9 (10.0)	
Immunosuppressant	75 (35.7)	17 (18.9)	
Biologics	84 (40.0)	58 (64.4)	
Perianal disease, n (%)	85 (40.5)	29 (32.2)	0.177
Endoscopic disease activity, n (%)	156 (74.3)	69 (76.7)	0.663

IQR: interquartile range, ASA: aminosalicylic acid

*P* values were determined by Mann-Whitney U test for continuous variables, the chi-square test and fisher exact test for categorical variables

### Comparison of laboratory indicators between patients with endoscopic activity and those in endoscopic remission

**Fig. 1:**
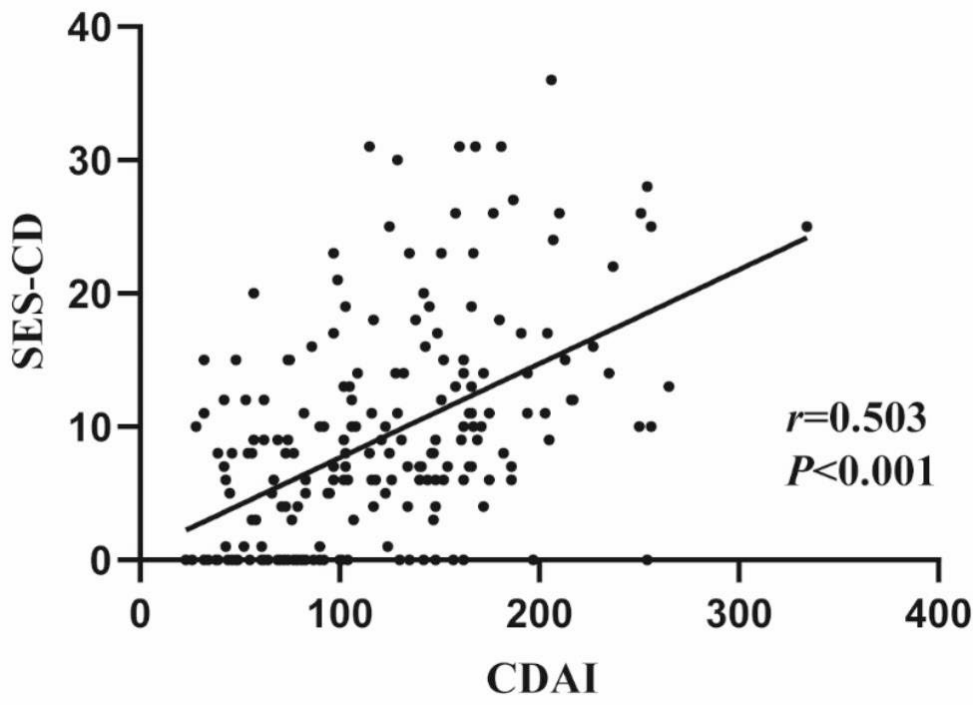
Scatter diagram of correlation between the CDAI and the SES-CD. Spearman’s rank order correlation coefficient 0.503 (*P* < 0.001)

We identified 156 patients who presented endoscopic disease activity in the training cohort. Compared with those with endoscopic disease remission, patients suffering from a flare exhibited significant difference in all of these indices except white blood cells (WBC). (Table 2). We found that the group of endoscopic remission had significantly higher HB (140 g/L vs. 121.5 g/L), lymphocytes (1.52 vs. 1.2 × 10^9^/L) and ALB (41.4 vs. 37.4 g/L) (*P* < 0.05). For patients with endoscopic activity, neutrophils (3.57 vs. 3.17 × 10^9^/L), PLT (247.5 vs. 205 × 10^9^/L), CRP (13.9 vs. 0.77 mg/L), NLR (3.01 vs. 2.10), PLpR (11.5 vs. 6.27), PLT/ALB (6.38 vs. 4.88), CRP/ALB (0.36 vs. 0.02) were significantly higher than those in endoscopic remission (*P* < 0.05). Concerning the CDAI score, the median score in the remission group and activity group in the training cohort was 77 and 130, respectively, which were moderately correlated with SES-CD (*r* = 0.503, *P* < 0.001) (Fig. 1). Similar differences were also observed in the test cohort.

**Table 2:** Comparison of laboratory indicators between endoscopic remission group and endoscopic activity group in Training and Test Cohort

Variable	Training Cohort (n = 210)			Test Cohort (n = 90)		
	Endoscopic Remission (n = 54)	Endoscopic Activity (n = 156)	*P* value	Endoscopic Remission (n = 21)	Endoscopic Activity (n = 69)	*P* value
CDAI	77 (49-100.25)	130 (92-166.75)	< 0.05	70 (40.5–94.5)	149 (99–203)	< 0.05
HB (g/L)	140 (122-150.25)	121.5 (108.3-138.8)	< 0.05	141 (112.5–141)	123 (105–135)	< 0.05
WBC(×10^9^/L)	5.41 (4.28–6.46)	5.5 (4.27–7.28)	0.484	5.99 (4.56–6.67)	6.24 (5.2–8.43)	0.144
N (×10^9^/L)	3.17 (2.45–4.02)	3.57 (2.69–5.04)	< 0.05	3.05 (2.69–3.53)	4.18 (3.23–5.92)	< 0.05
L (×10^9^/L)	1.52 (1.1–2.3)	1.2 (0.86–1.57)	< 0.05	2.04 (1.34–2.6)	1.39 (0.99–1.92)	< 0.05
PLT (×10^9^/L)	205 (170.75–233.5)	247.5 (200-315.5)	< 0.05	211 (176.5-231.5)	273 (231–331)	< 0.05
CRP (mg/L)	0.77 (0.5–3.48)	13.9 (4.65–37.62)	< 0.05	1.21 (0.38–4.53)	9.47 (3.22–34.3)	< 0.05
ALB (g/L)	41.35 (38.38–44.1)	37.35 (33.2–41.8)	< 0.05	45.9 (42.8–47.7)	41.5 (36.2–44.8)	< 0.05
NLR	2.1 (1.38–3.27)	3.01 (2.06–4.78)	< 0.05	1.76 (1.11–2.41)	2.94 (2.12–4.79)	< 0.05
PLpR	6.27 (5.11–9.3)	11.5 (7-19.33)	< 0.05	5.57 (4.88–7.84)	12.96 (9.9-17.89)	< 0.05
PLT/ALB	4.88 (4.06–5.89)	6.38 (4.96–8.86)	< 0.05	4.6 (3.7–5.63)	7.48 (5.72–8.84)	< 0.05
CRP/ALB	0.02 (0.01–0.09)	0.36 (0.12–1.11)	< 0.05	0.03 (0.01–0.11)	0.26 (0.07–0.89)	< 0.05

CDAI: Crohn’s disease activity index; HB: hemoglobin; WBC: white blood cell; N: neutrophil; L: lymphocyte; PLT: platelet; CRP: C-reactive protein; ALB: albumin; NLR: neutrophil-to-lymphocyte ratio; PLpR: platelet-to-lymphocyte percentage ratio

Data are presented as median (IQR). *P* values were determined by Mann-Whitney U test for continuous variables.

### 3.3. Comprehensive index for detecting endoscopic disease activity

We detected endoscopic activity by drawing ROC curves based on clinical and laboratory indicators which were significant in factor analysis (Table 3). When we chose 2 as the cut-off value for SES-CD to differentiate between endoscopic remission and activity, the maximum areas under ROC curve (AUC) of CRP and CRP/ALB were 0.826 and 0827, followed by CDAI (0.769), PLpR (0.735), PLT/ALB (0.712). The rest AUCs are all less than 0.7. The critical values of each indicator were CRP (6.1 mg/L), CRP/ALB (0.09), CDAI (101.5), PLpR (10.16), PLT/ALB (6.0) according to the Youden index. In order to make the indicators more convenient to be used in clinical practice, we selected the integer values closest to the cut-off values of each indicator, which was easy to be widely used.

**Table 3:** Median (95% CI) of AUC, cut-off value (in terms of the maximized Youden index) for identifying endoscopic activity in Training Cohort

Variable	AUC	95% CI	Cut-off value	*P* value
HB (g/L)	0.676	0.596–0.756	137.5	< 0.05
N (×10^9^/L)	0.603	0.521–0.685	4.14	< 0.05
L (×10^9^/L)	0.660	0.576–0.744	1.42	< 0.05
PLT (×10^9^/L)	0.682	0.605–0.759	236	< 0.05
CRP (mg/L)	0.826	0.764–0.888	6.1	< 0.05
ALB (g/L)	0.686	0.610–0.763	36.15	< 0.05
NLR	0.690	0.611–0.769	1.79	< 0.05
PLpR	0.735	0.663–0.807	10.16	< 0.05
PLT/ALB	0.712	0.636–0.789	6.0	< 0.05
CRP/ALB	0.827	0.765–0.888	0.09	< 0.05
CDAI	0.769	0.698–0.841	101.5	< 0.05

HB: hemoglobin; N: neutrophil; L: lymphocyte; PLT: platelet; CRP: C-reactive protein; ALB: albumin; NLR: neutrophil-to-lymphocyte ratio; PLpR: platelet-to-lymphocyte percentage ratio; CDAI: Crohn’s disease activity index

The indices with *P* < 0.05 in factor analysis were enrolled in the binary multivariable logistic regression analysis. Considering the collinearity, we adopted the backward stepwise regression method. Ultimately when including CRP, CDAI and PLpR, the fit of the model is optimal. As shown in Table 4, higher PLpR (OR: 2.777 [95%CI: 1.175–6.502], *P* = 0.02), higher CRP (OR: 5.262 [95%CI: 2.359–11.734], *P* < 0.05) and higher CDAI (OR: 4.165 [95%CI: 1.908–9.096], *P* < 0.05) were positively related to the endoscopic activity.

**Table 4:** Multivariate Logistic Regression Model for prediction endoscopic disease activity

		B	SE	OR	95% CI	*P* value
PLpR	≤ 10^*^					
	> 10	1.021	0.439	2.777	1.175–6.502	< 0.05
CRP (mg/L)	≤ 5^*^					
	> 5	1.660	0.409	5.262	2.359–11.734	< 0.05
CDAI	≤ 100^*^					
	> 100	1.427	0.398	4.165	1.908–9.096	< 0.05

* represents the control group; SE: standard error; OR: odds ratio; CI: confidence interval

CRP: C-reactive protein; PLpR: platelet-to-lymphocyte percentage ratio; CDAI: Crohn’s disease activity index

**Fig. 2:**
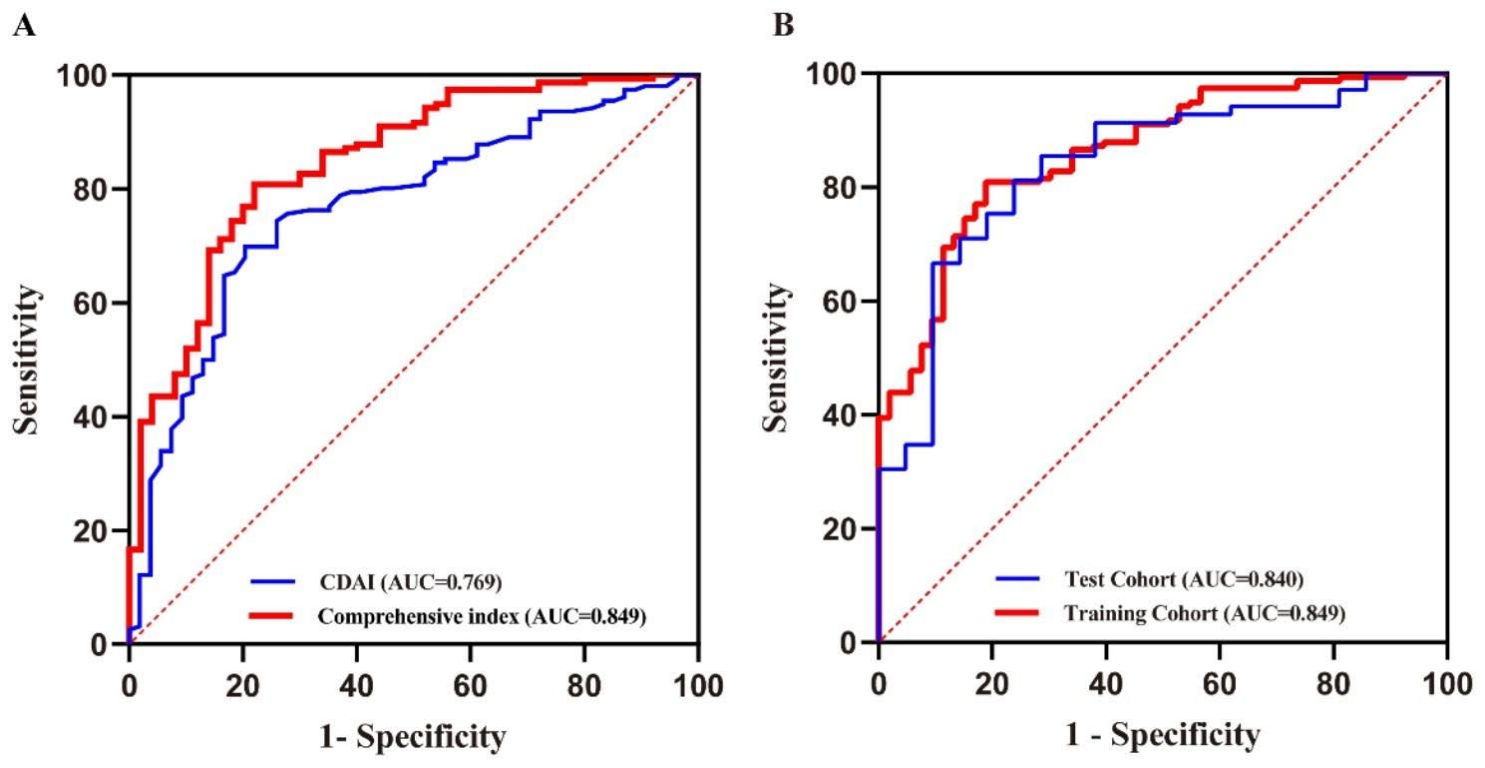
(**a**) ROC curves of the CDAI and the comprehensive index model (*P* = 0.002) for predicting the risk of endoscopic activity in the training cohort. (**b**) ROC curves for the comprehensive index in the training and test cohorts (*P* = 0.87) for predicting the risk of endoscopic activity

**Fig. 3:**
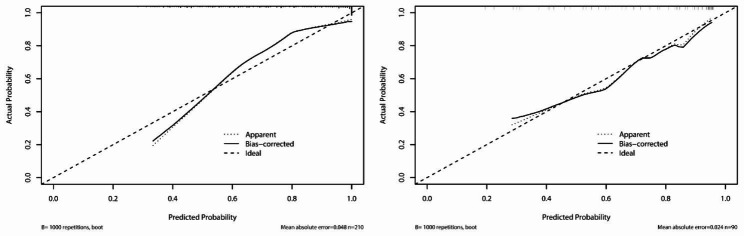
The calibration curves of the comprehensive index in the training and test cohorts. (a) The cali-bration curves for the comprehensive index in the training cohort (Brier score = 0.134) (b) and in the test cohort (Brier score = 0.128)

Subsequently, we combined three indicators to form a new comprehensive index. As shown in Fig. 2a, compared with CDAI, the AUC of the comprehensive index significantly increased (0.849 vs. 0.769, *P* < 0.05). The predictive performance of the comprehensive index in the training and test cohort was presented in Fig. 2b. The AUC of the model in the training cohort did not differ statistically from this in the test cohort (0.849 vs. 0.84, *P* = 0.87). Furthermore, the sensitivity (80.77% vs. 81.16%), specificity (79.63% vs. 76.19%), positive predictive value (PPV) (0.808 vs. 0.918), negative predictive value (NPV) (0.796 vs. 0.552) and accuracy (0.805 vs. 0.8) of the model for evaluating endoscopic activity in the training and test cohort were shown in Table 5. The comprehensive index also showed good and similar calibration curve in training and test cohort, indicating high accuracy (Brier score: 0.134 vs. 0.128) (Fig. 3a and b). In order to investigate the difference of the predictive value of single and comprehensive index, these indicators were compared both with one another and with SES-CD using the Kappa statistic (Table 6). The result showed that the comprehensive index performed the best at predicting endoscopic activity (*κ* = 0.542), followed by CRP (0.446), then CDAI (0.380), and finally PLpR (0.299).

**Table 5:** Predictive Performances of the comprehensive index in the Training Cohort and Test Cohort

Variable	AUC	Sensitivity (%)	Specificity (%)	PPV	NPV	Accuracy
Training Cohort						
Comprehensive index^#^	0.849 (0.81–0.916)	80.77	79.63	0.808	0.796	0.805
Test Cohort						
Comprehensive index	0.84 (0.744–0.936)	81.16	76.19	0.918	0.552	0.8

^#^Comprehensive index: the predicted probability of endoscopic disease activity based on the combination of CDAI, CRP and PLpR, and it was calculated using binary logistic regression model (ln(p/1-p) = 0.013*CDAI + 0.072*CRP + 0.067*PLpR-1.650)).

PPV: positive predictive value; NPV: negative predictive value

**Table 6:** Agreement between indices and each other, and with SES-CD ≥ 3

Model	Pred	Comprehensive index		CRP		PLpR		CDAI		SES-CD	
		I	A	I	A	I	A	I	A	I	A
Comprehensive index	I			60	12	62	10	62	10	43	29
	A			23	115	45	93	26	112	11	127
	κ			**0.663**		**0.473**		**0.649**		**0.542**	
CRP	I					62	20	53	29	42	40
	A					45	83	35	93	12	116
	κ					**0.405**		**0.368**		**0.446**	
PLpR	I							60	47	44	63
	A							28	75	10	93
	κ							**0.290**		**0.299**	
CDAI	I									41	47
	A									13	109
	κ									**0.380**	

κ = Kappa; A = active; I = inactive; Pred = predictor

CRP: C-reactive protein; PLpR: platelet-to-lymphocyte percentage ratio; CDAI: Crohn’s disease activity index

### Predictive performance of the comprehensive index in subgroup analysis

**Fig. 4:**
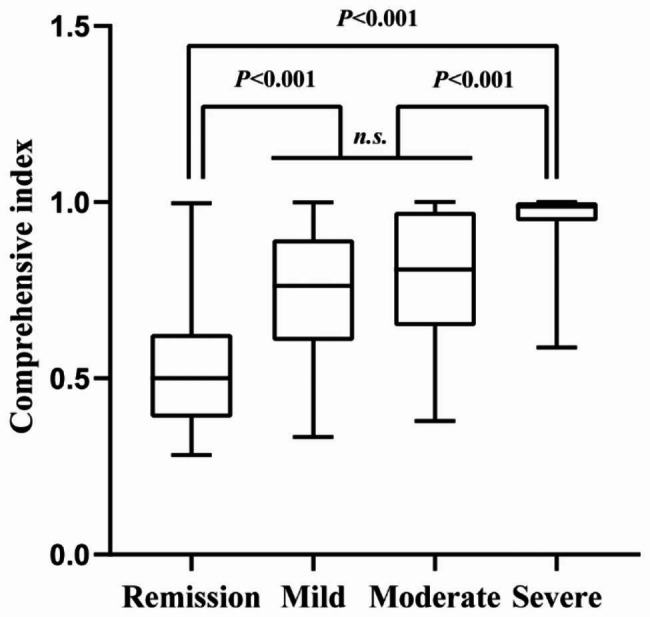
Median and interquartile of the comprehensive index in patients with different endoscopic activity; n.s., not statistically significant

**Fig. 5:**
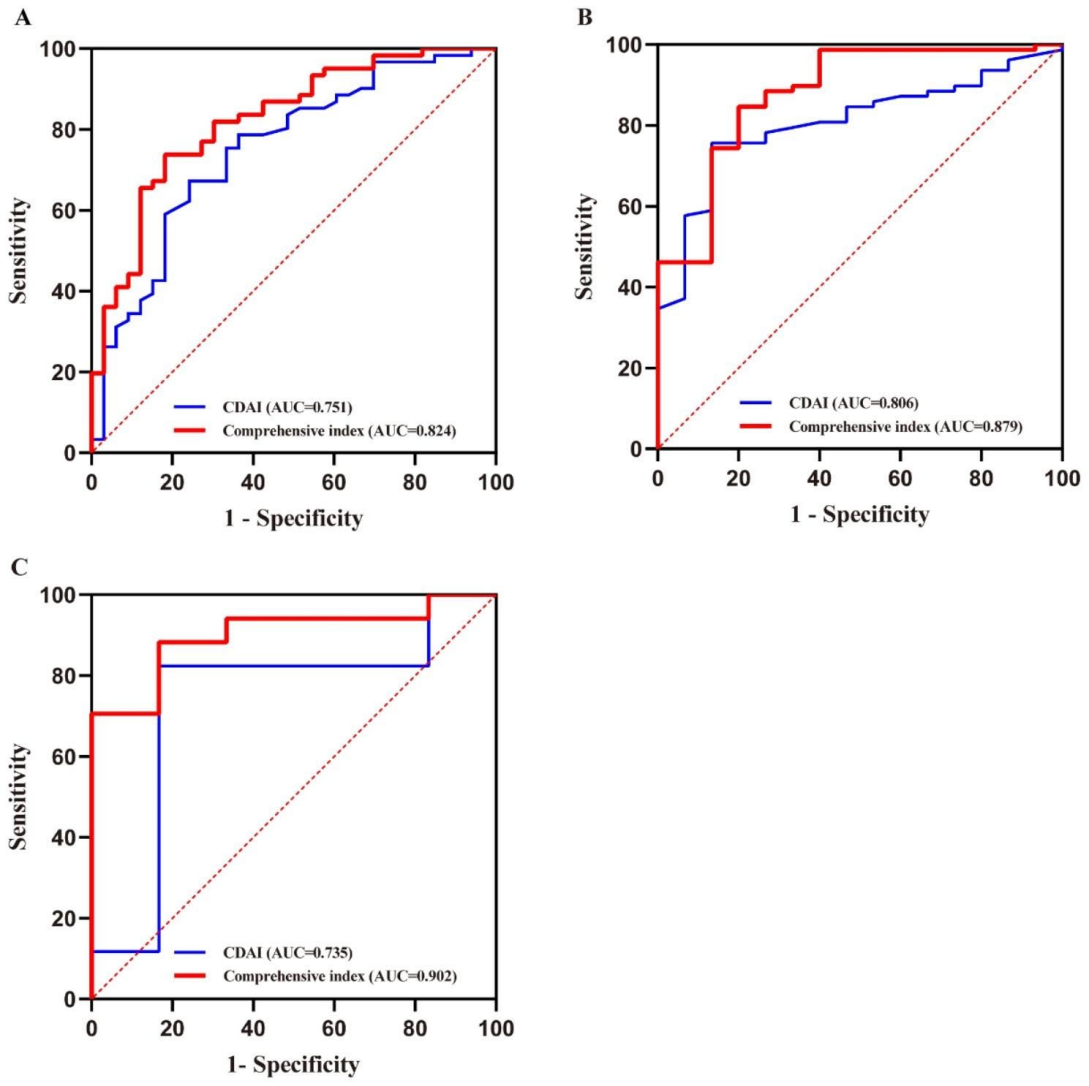
ROC Curve of CDAI and combination model for identifying endoscopic activity in patients with different CD behaviours. (**a**) patients with inflammatory behaviour (*P* = 0.028), (**b**) patients with stricturing behaviour (*P* = 0.132), and (**c**) patients with penetrating behaviour (*P* = 0.107)

In order to evaluate the role of comprehensive index in endoscopically disease severity, we performed a subgroup analysis and revealed that no statistically significant difference was found only between the endoscopic mild activity group and moderate group (Fig. 4). Regarding CD phenotype, we further developed a subgroup analysis. The comprehensive index had a higher AUC than CDAI in patients with inflammatory phenotype (Fig. 5a.824 vs. 0.751, *P* < 0.05). Whereas it showed no significant difference to CDAI in patients with stricturing and penetrating phenotype (Fig. 5b **and c**).

## Disscussion

The absence of endoscopic disease activity in patients with CD is associated with better clinical outcomes and is considered as the long-term targets [8]. However, frequent endoscopic examination is neither acceptable nor suitable for patients [11]. Therefore, it is crucial to explore an accurate and convenient non-invasive marker to assess endoscopic disease activity. In this retrospective multicenter study, we demonstrated that the comprehensive index including CDAI, PLpR and CRP showed the best performance characteristic in predicting endoscopic disease activity in ileocolic CD. The AUC was 0.849 and the sensitivity, PPV, NPV and accuracy were all superior to any of the index alone.

In our study, SES-CD was used to evaluate the endoscopic disease activity and CDAI was estimated for clinical severity. According to the published studies, SES-CD cut-off value for defining endoscopic remission were empirically chosen by researchers and has not been standardized yet. In our study, we chose relatively strict definition of SES-CD < 3 for endoscopic remission because past study has demonstrated that patients with complete endoscopic remission had fewer surgeries, hospitalizations, and a decreased risk of treatment failure than patients with partial endoscopic remission [26]. It has long been known that CDAI was commonly used in clinical practice to monitor symptom severity. Given the fact that CDAI index were subjective and could be influenced by various disease factors, it is not surprising that several studies have demonstrated the poor correlation between CDAI and endoscopic disease activity during the past decades [27–29]. Consistently, we observed that 30.1% of patients with endoscopic activity showed clinical remission, whereas 24.1% patients with endoscopic remission were in apparent clinical activity. Despite the existence of the population mentioned above, our study still showed the relatively moderate correlation (*r* = 0.503) between CDAI and SES-CD (Fig. 1). One possible explanation for this finding could be that inpatients might had more severe clinical symptoms and ileocolic inflammation.

In light of the fact that clinical symptom alone cannot clearly assess the endoscopic activity, previous studies took a strategy that using a comprehensive index which contained clinical parameters and noninvasive indices as a surrogate measurement [12, 30]. For instance, Langhorst et al. observed that combination of CDAI, CRP and stool biomarkers could substantially improve the accuracy of predicting endoscopic activity [30]. In addition to CDAI, some researchers also combined Harvey-Bradshaw index (HBI) or patient-reported outcomes (PROs) with certain noninvasive indices to improve the diagnostic accuracy of endoscopic activity in patients with CD [31, 32]. In 2020, Morris et al. proposed a novel model called PRO + that combined PRO, FC and hsCRP. They demonstrated that the PRO + model was superior to any of the single indicator for predicting disease activity [32]. However, in our study, the correlation between PRO and SESCD was worse than that between CDAI and SESCD (Figure S1). Considering that HBI and PRO were also simplified scores which lack of systematic evaluation, we ultimately decided to combine noninvasive indicators on the basis of CDAI to monitor endoscopic activity.

To date, fecal and blood indicators are the most widely used noninvasive indices to offer objective assessment of disease activity in patients with CD. Although fecal indicators, such as calprotectin and lactoferrin are well-established stool biomarkers of endoscopic activity [33], their lack of convenient, costly and compliance to patients limits their clinical value, especially in primary medical institutions. Therefore, the blood indices were chosen as objective indicators for evaluating the disease activity in present study. We screened blood indices from the published literature and ultimately enrolled CRP and PLpR by logistic regression to assess endoscopic activity. Of note, our study is the first time to simultaneously enroll and analysis the previously validated blood indicators. The result demonstrated that the composite index including CDAI, PLpR and CRP showed the best performance characteristic in predicting endoscopic disease activity. Interestingly, compared with fecal calprotectin which is a common, validated and non-invasive marker in CD patients, our model also has a relatively good ability to predict endoscopic activity [34]. As we all know, CRP is by far the most widely investigated serum index in evaluating disease activity and predicting therapeutic outcomes in clinical practice. Tremendous studies had demonstrated the capacity of CRP elevation for detecting mucosal inflammation [34, 35]. In a cross-sectional study, Yarur et al. showed that the AUC, sensitivity and specificity of CRP were separately 0.75, 69% and 62% in the evaluation of endoscopic disease activity [36], which is in consistence with our outcomes. PLpR (Platelet count (109/L) / Lymphocyte percentage (%)) was first proposed by Rirong Chen as an indirect indicator to predict endoscopic activity in 2020 [23]. Their study showed that PLpR performed good characteristic in evaluating endoscopic activity, with an AUC of 0.785 (95%CI 0.784–0.787) and a cut-off value of 11.51. Furthermore, authors also observed that the accuracy of predicting endoscopic activity could be further improved when combined PLpR with CRP, which was also in agreement with our outcome that the AUC of the composite index combined CDAI with PLpR and CRP is superior to the combination of CDAI and CRP.

Considering that there was statistical difference between disease behaviours and endoscopic disease activity in our cohort, we performed a subgroup analysis on the basis of the phenotypes and found that the AUC of the composite index was higher than CDAI significantly (0.824 vs. 0.751, *P* < 0.05) in inflammatory phenotype (B1), while they were no statistical significance in stricturing (B2) and penetrating (B3) phenotype. One possible explanation for this phenomenon might be the difference of CRP levels and clinical severity in each phenotype. CRP is an acute-phase protein and rises frequently at the early stage in CD [37], and many studies showed that the higher proportion of complicated disease phenotypes would occur with the duration of the course of CD [6, 38]. Bo Shen et al. showed that inflammatory CD mainly presented with mild diarrhea and/or abdominal pain, whereas fibrostenotic CD presented with severe obstructive symptoms, fistulizing CD manifested a range of severe symptoms caused by intestinal perforation [39]. Although the statistical difference was not observed between the comprehensive index and CDAI in B2 and B3 subgroups, the rising trend could still be obviously found. In the follow up study, a larger sample sizes could be enrolled for further validation.

The main limitations of the present study include its retrospective design and relatively small number of participants. To reduce the bias occurred in data collection and analysis, doctors in charge of endoscopic scoring were not aware of other data. Although external validation was performed, the sample size of the validation group was relatively small. Further studies involving a large-scale sample in multicenter validation awaits to be conducted. Considering the fact that SES-CD scored by colonoscopy is the most widely used endoscopic scoring system in evaluating disease activity, we chose it as the gold standard. However, it could not assess the condition of small bowel CD accurately [40], which led to the unclear effects in the intestinal segment. In addition, recent studies had shown that some validated and up-to-date biomarkers such as fecal calprotectin, vitamin D [41], prealbumin [42] and cytokines [43] also could assess the endoscopic activity significantly which need our further validation in future study.

## Conclusion

The combination of CDAI, CRP and PLpR provides physicians with a novel noninvasive, convenient and economical measurement which can accurately evaluate the disease activity and highly correlated with SES-CD, especially in patients with inflammatory phenotype. Our findings suggest the potential of applying the surrogate indicators to discriminating disease activity in ileocolic CD patients. It could be used as a clinical decision support tool for the management of patients with CD.

### Electronic supplementary material

Below is the link to the electronic supplementary material.

Supplementary Material 1

## Data Availability

The raw data supporting the conclusion of this article will be made available by the correspondence author via email shou@yzu.edu.cn, without undue reservation.
