# Supplementary material for: Combined use of CDAI and blood indices for assessing endoscopic activity in ileocolic Crohn’s disease
Source: BMC Gastroenterol. 2023 Sep 28;23:337. doi: 10.1186/s12876-023-02968-0 (PMC10540500; doi:10.1186/s12876-023-02968-0)
Supplement: Supplementary file 1 — Supplementary Material 1 [file 12876_2023_2968_MOESM1_ESM.docx]

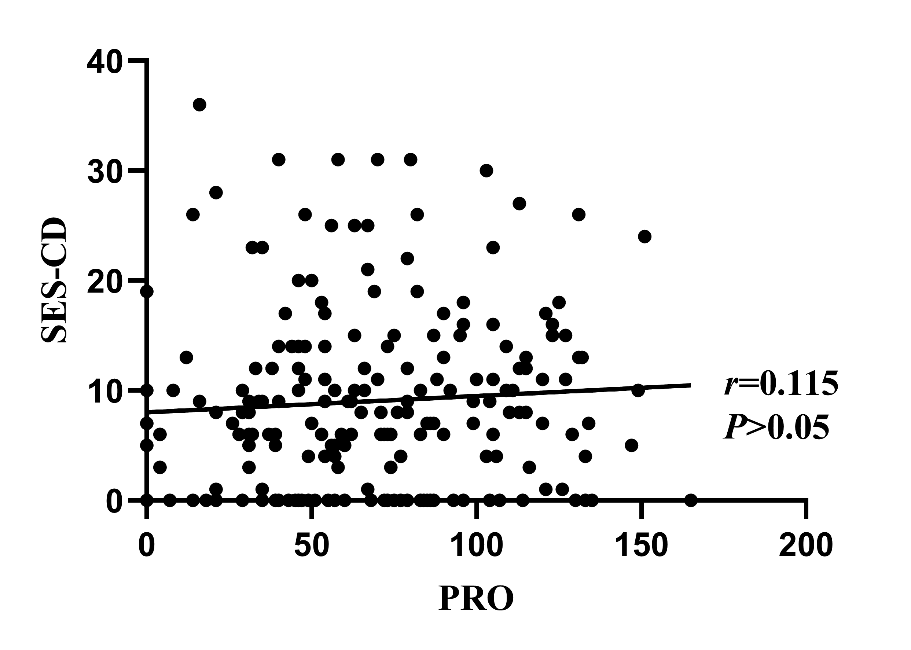


**Figure S1** Scatter diagram of correlation between the PRO and the SES-CD. Spearman's rank order correlation coefficient 0.115 (*P* > 0.05)
